# PROTECTOR / FIRE‑10: study protocol for a prospective, randomized, open-label, multicenter phase III trial to investigate the efficacy of preoperative systemic therapy in advanced colon cancer

**DOI:** 10.1186/s12885-026-15682-3

**Published:** 2026-03-20

**Authors:** Robert Siegel, Luca Dittrich, Julius Plewe, Timm Denecke, Jakob Leonhardi, Andrea Tannapfel, Anke Reinacher-Schick, Nathanael Raschzok, Igor Maximilian Sauer, Johannes Lauscher, Sylvie Lorenzen, Thorsten Götze, Ivan Jelas, Arndt Stahler, Annika Kurreck, Alexej Ballhausen, Benjamin Nils Ostendorf, Oliver Haase, Johann Pratschke, Sebastian Stintzing, Dominik Paul Modest

**Affiliations:** 1 Department of Surgery, Campus Charité Mitte | Campus Virchow Klinikum, Charité – Universitätsmedizin Berlin, corporate member of Freie Universität Berlin and Humboldt-Universität zu Berlin, Berlin, Germany; 2 Department of Diagnostic and Interventional Radiology, University Medical Center Leipzig, Leipzig, Germany; 3 Institute of Pathology, Ruhr-Universität Bochum, Bochum, Germany; 4 Department of Hematology and Oncology, Katholisches Klinikum Bochum, Universitätsklinik der Ruhr-Universität Bochum, Bochum, Germany; 5 Berlin Institute of Health at Charité – Universitätsmedizin Berlin, BIH Academy, Clinician Scientist Program, Berlin, Germany; 6 Department of General and Abdominal Surgery, Campus Benjamin Franklin, Charité – Universitätsmedizin Berlin, corporate member of Freie Universität Berlin and Humboldt-Universität zu Berlin , Berlin, Germany; 7 Department of Hematology and Oncology, Technical University of Munich, Munich, Germany; 8 Krankenhaus Nordwest - University Cancer Center Frankfurt, Frankfurt, Germany; 9 Department of Hematology, Oncology, and Cancer Immunology, Charité – Universitätsmedizin Berlin, corporate member of Freie Universität Berlin and Humboldt-Universität zu Berlin, Berlin, Germany; 10 NCT- National Center for Tumor Diseases Berlin, Berlin, Germany

**Keywords:** Colon cancer, Colorectal cancer, Preoperative chemotherapy, Neoadjuvant therapy, Colorectal surgery

## Abstract

**Background:**

Standard of care for non-metastatic colon cancer is surgery followed by stage-guided adjuvant therapy and/or structured follow-up. Upfront surgery in resectable colon cancer is irrespective of local T/N stage, whereas adjuvant systemic therapy is recommended according to pathological staging. The role of neoadjuvant chemotherapy remains unclear. Whereas perioperative systemic therapy in colon cancer seems to be safe and may lead to pathologic downstaging, evidence on improved survival and quality of life remains scarce. The PROTECTOR / FIRE‑10 trial aims to generate evidence that perioperative systemic therapy improves survival without compromising quality of life in locally advanced, mismatch-repair proficient colon cancer patients.

**Methods and design:**

Open-label, randomized, controlled, multicenter, phase III study with two parallel arms. Patients with locally advanced colon or upper rectal cancer staged cT3-4 and/or cN+ are randomized in a 2:1 fashion (favoring preoperative therapy) to investigate the efficacy, patient reported quality of life, and safety of preoperative therapy followed by surgery (Arm A) versus direct surgery followed by non-study specific stage-guided adjuvant therapy (Arm B). Stratification during randomization will be performed according to the following parameters: Fit for mFOLFOXIRI vs. mFOLFOX/CAPOX vs. 80%-mFOLFOX/CAPOX, ECOG 0 vs. ECOG 1-2, and left-sided primary vs. right-sided primary tumor. Only patients with confirmed mismatch-repair proficient and/or microsatellite stable tumor can be included.

Preoperative treatment in Arm A is performed for a maximum of 6 biweekly cycles of FOLFOX/FOLFOXIRI or for a maximum of 4 triweekly cycles CAPOX (i.e., appr. 12 weeks). Patients in both arms should undergo quality-controlled surgery of the primary tumor, performed as complete mesocolic excision.

Patients will be followed up with regard to relapse, survival and if applicable subsequent anti-cancer treatments until death or for at least 5 years after randomization, whichever date is earlier.

**Discussion:**

The PROTECTOR / FIRE‑10 trial compares preoperative systemic therapy to upfront surgery (with stage-guided adjuvant therapy) in patients with locally advanced colon cancer.

**Trial registration:**

This study is registered with clinicaltrials.gov (NCT06899477) and EudraCT (2023-508076-11-00).

**Supplementary Information:**

The online version contains supplementary material available at 10.1186/s12885-026-15682-3.

## Background

Colorectal cancer is the third most common cancer diagnosed in women and men, it was the fourth-leading cause of cancer death in both men and women younger than 50 years in the late-1990s but is now first in men and second in women [1]. In Germany, approximately 55,000 to 60,000 new cases are diagnosed annually [2]. Colon cancer accounts for two thirds of all colorectal cancer cases.

In patients with colon or upper rectal cancer, irrespective of the assumed T/N-stage, the standard of care in the absence of metastases is surgery, which is followed by stage-guided adjuvant therapy and/or structured follow-up [3]. Adjuvant therapy is clearly indicated in UICC stage III patients (nodal positive) and also recommended in some patients with UICC II (T3-4, N0 tumors) with respective risk criteria (T4 tumors, emergency surgery, perforation, insufficient lymph node harvest etc.) [3, 4].

The strategy of neoadjuvant or perioperative systemic therapy represents an emerging concept in solid tumors and is already a standard of care in some gastrointestinal cancers such as advanced gastric and esophageal junction cancer, and notably in advanced lower rectal cancer [5–8]. Concepts addressing the lower rectal cancer, biologically similar to colon cancer, include total neoadjuvant therapy (TNT) as a new standard of care [7, 8]. Potential benefits of neoadjuvant therapy for patients, contributing to improved long-term outcome, include:

- Population effect: nearly 100% exposition to chemotherapy vs. only those patients that are fit enough to receive chemotherapy after resection.
- Earlier treatment of micrometastases.
- Downstaging of the primary tumor, enabling easier resections.
- Pre-habilitation before radical surgery.

In locally advanced colon cancer, two phase III trials have investigated perioperative treatment with 6/12 weeks of systemic therapy before surgery, in both cases doublet therapy with FOLFOX/CAPOX. The FOXTROT trial was first presented at the 2019 ASCO congress with lacking anticipated event rates and was consecutively rated “negative” with a borderline-significant *p*-value. This was despite exactly hitting the predefined effect size. In summary, FOXTROT suggested that perioperative therapy in colon cancer is safe and might be beneficial in terms of disease-free survival and overall survival [9].

The OPTICAL trial investigated perioperative therapy with 3 months of FOLFOX/CAPOX before and after surgery as experimental arm and reported a trend towards better disease-free survival, that was comparable to the effect size in FOXTROT together with a significant effect on overall survival (secondary endpoint) [10].

In order to further develop the encouraging signals of the FOXTROT and OPTICAL trial and the currently implemented TNT in rectal cancer into a new standard of care for colon cancer, our study will include necessary modifications in the design as follows:

- Exclusion of dMMR/MSI tumors as these derived no benefit in the FOXTROT and OPTICAL trial [9, 10].
- Escalation of the treatment duration from 6 to 12 weeks in accordance with current concepts in rectal cancer to ensure more efficacy.
- Option to escalate therapy regimens (i.e., mFOLFOXIRI) [7, 8].
- Surveillance of T- and N- stages in the standard arm to ensure that the frequency of over-staged tumors in the trial can be controlled and does not affect the necessary event-size.

Together, with these modifications, the PROTECTOR / FIRE‑10 study will refine the clinical and oncological benefits of using preoperative systemic therapy in advanced colon cancer.

## Methods/design

### Aim of the study

The main objective of this randomized multicenter trial is to generate further evidence that preoperative chemotherapy improves disease-free survival in patients with advanced colon or upper rectal cancer.

### Study setting and trial population

PROTECTOR / FIRE‑10 is an investigator-initiated prospective randomized open-label multicenter phase III trial with two parallel study groups. The trial is organized by the Charité - Universitätsmedizin through the German Alliance “Arbeitsgemeinschaft Internistische Onkologie (AIO)” for Clinical Cancer Trials, sponsored by the Deutsche Krebshilfe (German Cancer Aid).

Patients with colon or upper rectal (above 12 cm form anal verge) cancer staged cT3-4 and/or cN + and eligible for curative surgery will be included in the trial.

**Fig. 1:**
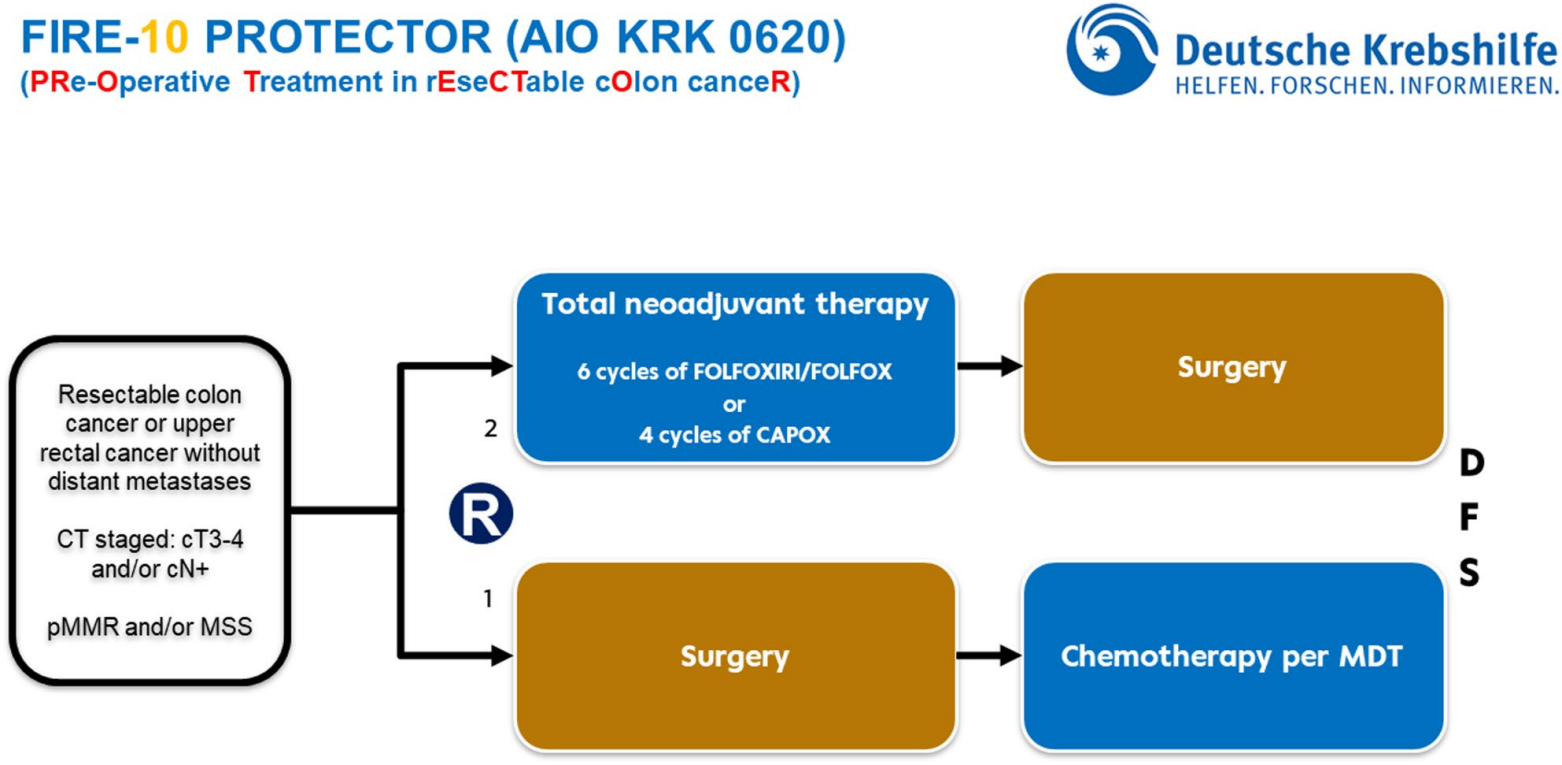
Illustration of Trial Design. (CT = computed tomography; R = randomization; MDT = multidisciplinary tumor board; DFS = disease free survival)

See Fig. 1 for an illustration of the study design.

### Sample size

Based on the best available evidence described above [9, 10], 2-year DFS rate is 70% in the straight to surgery group and we hypothesize that DFS rate will be 78% in the neoadjuvant treatment followed by surgery group. To demonstrate an increase of 8% points in 2-year DFS rate (hazard ratio = 0.696), it is required to observe 271 DFS events, with the recruitment of 714 (476:238 per arm) patients with an allocation ratio of 2:1 (experimental vs. standard). These calculations assume that the DFS time follows a piecewise exponential distribution with two strata within the maximum follow up time ([0–2] years [2–8], years). For the first stratum, we assume that DFS rate will be 70% after 2 years in the control group. For the second stratum, we expect a level-off effect to a plateau of 60% DFS rate after 8 years. The sample size was calculated with 2-sided 5% level of significance, 80% power, with 5-year recruitment and 5-year follow-up periods and 5% drop-out in the first two years. The event rate in the straight to surgery group will be monitored annually by the independent data monitoring and ethics committee to ensure the baseline assumptions are appropriate. An interim analysis for efficacy will not be conducted. Given that an inferiority is unlikely with the background of TNT concepts in rectal cancer and previous trials and the increased sample size would impact on the feasibility of recruitment, interim analyses will solely focus on safety.

### Inclusion criteria

Participants are eligible to be included in the trial only if all of the following criteria apply at time of enrollment:

1. Patient’s signed informed consent.
2. Patient’s age ≥ 18 years at the time of signing the informed consent.
3. Histologically confirmed adenocarcinoma of the colon or upper rectum.
4. Confirmed mismatch-repair proficient and/or microsatellite stable tumor. Both immunohistochemistry and PCR can be used for diagnosis.
5. Intent for curative surgery.
6. Predicted (clinical) T3 or T4 stage and or nodal positivity (N+) assessed by the local study team based on computed tomography and/or magnetic resonance imaging of the abdomen and pelvis. The imaging criteria were adjusted to achieve a low false positive rate.
  T3-4 defined as unequivocal signs of invasion of surrounding tissue structures or organs
  N+ defined as regional (within drainage territory of the primary site) lymph node(s) without fat hilus or occupying calcifications enlarged to a short/long axis diameter of ≥1/1.5 cm (for ileocecal region ≥ 1.3/1.8 cm)
7. Absence of clear distant metastases assessed by the investigator based on respective routine evaluations within 6 weeks prior to inclusion into the trial (Preferred: computed tomography of thorax and abdomen. Alternatively, magnetic resonance images, FDG-PET, sonography and x-rays might be used for assessment).
8. Absence of significant active wound healing including severe chronic non-healing wounds, ulcerous lesions or untreated bone fracture.
9. ECOG performance status 0-2.
10. Adequate bone marrow, hepatic and renal organ function, defined by the following laboratory test results:
  Absolute neutrophil count ≥ 1.5 x 109/L (1,500/µL).
  Hemoglobin ≥ 80 g/L (8 g/dL) with or without transfusion.
  Platelet count ≥ 100 x109/L (100,000/µL) without transfusion.
  Total serum bilirubin of ≤ 1.5 x upper limit of normal (ULN).
  Aspartate aminotransferase (AST) ≤ 3.0 × ULN.
  Calculated glomerular filtration rate (GFR) according to Cockcroft-Gault formula or according to MDRD ≥ 50 mL/min or serum creatinine ≤ 1.5 x ULN.
11. Patients without anticoagulation need to present with an INR < 1.5 x ULN and PTT < 1.5 x ULN. Patient with prophylactic or therapeutic anticoagulation are allowed into the trial.
12. Proficient fluorouracil metabolism as defined:
  - Prior treatment with 5-FU or capecitabine without unusual toxicity or,
  - If tested, normal DPD deficiency test according to the standard of the study site, or
  - If tested, in patients with DPD deficiency test with a CPIC activity score of 1.0-1.5 fluoropyrimidine dosage should be reduced by 50% and patients are allowed into the trial.
13. For women of childbearing potential (WOCBP): negative pregnancy test within 7 days before treatment initiation and agreement to remain abstinent (refrain from heterosexual intercourse) or use contraceptive methods with a failure rate of < 1% per year during the treatment period and for at least 6 months after the last dose of study treatment.

For men: With female partners of childbearing potential, men must remain abstinent or use a condom plus an additional contraceptive method that together result in a failure rate of < 1% per year during the treatment period and for 6 months after the last dose of study treatment. Men must refrain from donating sperm during this same period.

With pregnant female partners, men must remain abstinent or use a condom during the treatment period and for 6 months after the last dose of study medication to avoid exposing the embryo.

## Exclusion criteria

Participants are excluded from this trial if any of the following criteria apply at time of enrollment:

1. Malignant intestinal obstruction or directly imminent obstruction as assessed by the local study team. Patients with treated and resolved obstruction are allowed into the trial.
2. Previous chemotherapy for colorectal cancer of any stage.
3. New York Heart Association Class III or greater heart failure by clinical judgement.
4. Myocardial infarction within 6 months prior to randomization; percutaneous transluminal coronary angioplasty (PTCA) with or without stenting within 6 months prior to randomization.
5. Unstable angina pectoris.
6. Unstable cardiac arrhythmia > grade 2 NCI CTCAE despite anti-arrhythmic therapy.
7. Ongoing toxicities > grade 2 NCI CTCAE, in particular peripheral neuropathy.
8. Active uncontrolled infection by investigator’s perspective.
9. Known hypersensitivity to 5-FU, folinic acid, capecitabine, irinotecan or oxaliplatin or to any of the other excipients listed in Sect. 6.1 of the corresponding SmPC.
10. Recent or concomitant treatment with brivudine.
11. Peripheral sensitive neuropathy with functional impairment (> grade 1 acc. to CTCAE version 5.0.
12. Simultaneous application of St. John’s Wort preparations.
13. Pernicious or other megaloblastic anemia caused by vitamin B12 deficiency.
14. Major surgical procedure, open biopsy, or significant traumatic injury within 21 days prior to randomization that may interfere with systemic therapy as judged by the investigator.
15. Any other disease, metabolic dysfunction, physical examination finding, or clinical laboratory finding that contraindicates the use of an investigational drug, may affect the interpretation of the results, or may render the patient at high risk from treatment complications, including but not limited to:
  Simultaneous application of live vaccines during treatment with irinotecan and for at least 6 months after the last dose.
  5-FU must not be given in combination with brivudin, sorivudin and analogues to patients homozygous for DPD and patients known with completely missing DPD activity.
  Severe diarrhea.
16. Medical history of malignant disease other than colorectal cancer with the following exceptions:
  patients who have been disease-free for at least three years before randomization
  patients with adequately treated and completely resected basal cell or squamous cell skin cancer, in situ cervical, breast or prostate cancer, stage I uterine cancer
  patients with any treated or untreated malignant disease that is associated with a 5-year survival prognosis of ≥ 90% at the time of inclusion (assessed and documented by the investigator) and does not require active therapy
17. Known alcohol or drug abuse.
18. Pregnant or breastfeeding females.
19. Participation in a clinical trial or experimental drug treatment within 28 days prior to potential inclusion in the clinical trial or within a period of 5 half-lives of the substances administered in a clinical trial or during an experimental drug treatment prior to potential inclusion in the clinical trial, depending on which period is longest, or simultaneous participation in another clinical trial while taking part in this clinical trial.
20. Patients depended on Sponsor, investigator or study site.
21. Patient committed to an institution by virtue of an order issued either by the judicial or the administrative authorities.
22. Limited legal capacity.

### Randomization and stratification

Screened and eligible patients will be allocated in a strictly concealed way by 2:1 randomization (intervention arm A vs. control arm B) block with variable block lengths randomization. During the randomization process stratification will be performed according to the following binary stratification variables:

- fit for mFOLFOXIRI vs. mFOLFOX/CAPOX vs. 80%-mFOLFOX/CAPOX
- ECOG 0 vs. ECOG 1–2
- left-sided primary vs. right-sided primary tumor (left-sided: splenic flexure and distal, right-sided: caecum to transverse colon)

Due to the nature of the intervention, blinding is not possible. However, given that the intervention in this trial are well established chemotherapy regimens, a center-driven bias caused by differing expertise appears highly unlikely and will not be compensated for.

### Primary endpoint

Disease-free survival (DFS) – defined as no surgery, no resection, incomplete resection, disease recurrence (new metastases or local relapse) and death from any cause from the time of randomization. In case of no surgery, no resection, or incomplete resection (defined as R2 resection = macroscopic tumor rest), the timepoint of the respective event will be set to two weeks after randomization to avoid time-bias in favor of the experimental/neoadjuvant therapy arm.

### Secondary endpoints

#### Efficacy

- Overall survival (OS)
- Tumor regression by Dworak score

#### Safety

- Type, incidence, severity, and causal relationship to active chemotherapy of non-serious adverse events and serious adverse events (severity evaluated according to CTCAE version 5.0)
- For surgical procedures the following assessments will be conducted: frequencies of infections, re-surgeries, anastomotic leaks. Further: days in hospital after resection, TNM-staging of resected specimen, R0/R1 resection rates, complication scores (Clavien-Dindo classification)

### Quality of life

- Quality of life (QoL) as assessed with the QoL questionnaire EQ-5D-5L

### Exploratory endpoints

- DFS, OS and Safety according to circulating tumor DNA at baseline (ctDNA positive vs. negative).
- DFS, OS and Safety according to molecular subgroups
- Documentation of post-study treatment anti-cancer therapies and their efficacy

### Experimental intervention

The study drug interventions to be used in arm A of this trial are mFOLFOX-6 or CAPOX or mFOLFOXIRI. A guidance is outlined in Table 1, however, at the discretion of the investigator, site specific modifications are permitted, e.g., additional 5-FU bolus, parallel leucovorin and oxaliplatin administration or variation of the infusion rates.

**Table 1:** Trial medication (guidance for administration)

Drug	Dose/Potency	Duration of administration***	Route of Administration	Day(s) of application
*mFOLFOX-6 regimen*:				
Oxaliplatin Leucovorin 5-FU*	85 mg/m² 400 mg/m² 400 mg/m^2^ bolus** 2400 mg/m²	2 h 0.5–2 h 2–5 min 46 h	IV Infusion IV Infusion IV Infusion IV Infusion	All at d1 of each 14- days cycle
*CAPOX regimen*:				
Oxaliplatin Capecitabin	130 mg/m² 1000 mg/m²	~ 3 h Swallow twice daily	IV Infusion *Oral*	d1 d1-d14 of each 21-days cycle
*mFOLFOXIRI regimen*:				
Oxaliplatin Irinotecan Leucovorin 5-FU*	85 mg/m² 150 mg/m^2^ 400 mg/m² 2400 mg/m²	2 h 90 min 0.5–2 h 46 h	IV Infusion IV Infusion IV Infusion IV Infusion	All at d1 of each 14-days cycle

(FOLFOX/FOLFOXIRI are given every 2 weeks as standard, CAPOX is given every 3 weeks as standard. CAPOX and FOLFOX may also start with 80% dosage of the indicated 100% dose level

* in DPD mutation carriers with a CPIC activity score of 1.0-1.5, 5-FU dosage should be reduced by 50%

** additional 400 mg/m2 bolus is permitted but not mandatory

*** infusion rates of chemotherapeutical components represent recommendations but might be modified according to local standards)

Given that fluoropyrimidines and oxaliplatin were used in the FOXTROT trial, their rationale in an extended preoperative treatment sequence appears reasonable. Further support of the use of these regimens (FOLFOX/CAPOX) is found in the neoadjuvant treatment strategies established in rectal cancer [7, 11]. Unlike FOLFOX/CAPOX, FOLFOXIRI was only evaluated in one relevant phase III trial- PRODIGE-23- as regimen addressing locally advanced rectal cancer. The main rationale for implementing FOLFOXIRI as neoadjuvant/preoperative therapy also in colon cancers may be that initial response presents an important component of the treatment goal in the context of high-risk tumors for R1 resection (as selected by this study protocol) [8, 9].

Trial interventions should be administered on day 1 of each cycle after all procedures/assessments have been completed. From the second cycle onwards, the chemotherapy may be administered up to 5 days after the scheduled day 1 of the respective cycles, due to administrative reasons with a minimum time span of 14/21 days (FOLFOX and FOLFOXIRI / CAPOX, respectively) between the cycles.

### Control intervention

The control arm will offer the current standard of care. For locally advanced colon and upper rectal cancer, standard of care is comprised of primary surgery followed by stage-guided adjuvant therapy [3, 4]. Surgical resection of the primary tumor should be performed as complete mesocolic excision to ensure a surgically radical approach and to minimize a potential bias. Non-complete mesocolic excisions will not represent a protocol deviation, but must be documented in the corresponding clinical report forms. The trial steering committee will assess the surgical resection strategies (open surgery, laparoscopic and robotic approaches) within the trial site selection process. Furthermore, central assessment of resected specimen will provide an additional control of surgical quality.

### Structured follow-up

There will be a structured follow-up for at least 60 months after randomization/treatment, for which several assessments will be necessary. After the structured follow-up, patients will be monitored for DFS and OS in the survival follow-up. The suggested follow-up regimen is shown in Table 2. A deviation by one month is accepted. In case of CT/MRI assessments at any time-point, the sonography of the abdomen might be cancelled at the discretion of the investigator.

**Table 2:** Structured follow-up regimen

Investigation	Months in the follow-up (after surgery)							
	3	6	12	18	24	36	48	60
Patient history, anamnesis	x	x	x	x	x	x	x	x
Clinical examination	x	x	x	x	x	x	x	x
CEA assessment	x	x	x	x	x	x	x	x
CT (thorax/abdomen/pelvis)					x			
Abdominal ultrasound		x	x	x	x	x	x	x
Colonoscopy			x					

### Statistical analyses

#### Primary analysis

The null hypothesis to be tested in confirmatory analysis states that the hazard ratio for DFS comparing intervention versus control equals 1. This hypothesis will be tested by means of piecewise exponential model, by dividing the maximum follow-up time in 2 strata (0–2 years, 2–8 years), assuming the hazard is approximately equal in each time interval. This regression will be adjusted for the strata for randomization mentioned above. The two-sided significance level is set to 0.05. The primary analysis will be conducted based on the full analysis set which is defined as the intention to treat population. In a survival analysis setting, missing values are treated as non-informative censoring values so there is no need for imputation. 

#### Secondary analyses

As a sensitivity analysis to the primary efficacy analysis, the same test will be repeated on the per-protocol population including all patients without major protocol violations. As another sensitivity analysis, the primary hypothesis will also be evaluated by computing the restricted mean survival at 5 years. Event probabilities of DFS will be estimated by Kaplan-Meier-Curves. The above analyses will be repeated for overall survival. The influence of treatments received after the period of intervention on DFS and OS will be assessed. Specific post-study treatments will be included in the piecewise exponential model as time-dependent explanatory variables. Longitudinal models will be fitted to examine the evolution over time of the two arms and to test potential differences between them. Circulating tumor DNA, tumor markers and molecular information as well as quality of life/ patient reported outcomes will be evaluated exploratory.

The intent-to-treat population includes all randomized patients. Treatment assignment is based on the randomized treatment (primary population). The ITT population is the primary population for the description of the patient and treatment characteristics and is used for the primary efficacy analysis. The per-protocol (PP) population will include all randomized patients fulfilling the inclusion and exclusion criteria and who received at least one cycle of active treatment with at least one documented structured follow-up visits (arm A) or with at least one documented follow-up visit (arm B). Treatment assignment is based on the treatment actually received. Patients with major protocol violations will be excluded.

### Safety analyses

Safety analysis will be performed in the safety set equivalent to the full-analysis set, specified above. Absolute and relative frequencies as well as unbiased event-rate estimates (Kaplan-Meier, Empirical cumulative incidence) of AEs, SAEs, event rates of grade 3 and 4 toxicities (NCI-CTCAE v5.0) and abnormal laboratory values/ increase/decrease between treatment arms will be reported at different time points together with 95%-confidence intervals. Safety of surgery is measured by complication scores (Clavien Dindo), R0/R1 resection rates, length of hospital stay, repeated operations due to complications, post-surgical infections, and anastomotic leaks. All analyses will be done using validated statistical software. The safety population for chemotherapy related toxicities comprises all patients who received at least one administration of FOLFOXIRI/FOLFOX-6 or CAPOX after randomization.

A Data and Safety Monitoring Board will oversee the study, conducting interim safety reviews and ensuring protocol adherence. Additionally, a Surgical steering committee has been established.

## Discussion

Patients with locally advanced colon cancer, including the upper third of the rectum, will usually proceed directly to surgery in the absence of metastases. After surgery, adjuvant therapy is stratified by tumor stage and nodal involvement. Whereas nodal positive patients should receive three to six months of fluoropyrimidines plus oxaliplatin (CAPOX, FOLFOX), more individualized decisions, also based on mismatch repair status and clinical factors are made in stage UICC II patients (T3 or T4 without lymph node involvement). These decisions may include watch and wait up to oxaliplatin-based therapy [3, 12–15].

Both the FOXTROT and the OPTICAL trial investigated perioperative treatment with 6/12 weeks of systemic therapy before surgery in locally advanced colon cancer patients, in both cases doublet therapy with FOLFOX/CAPOX [9, 10]. The results of the FOXTROT trial suggested that perioperative therapy in colon cancer is safe and might be beneficial in terms of disease-free survival and overall survival [9]. The OPTICAL trial investigated perioperative therapy with 3 months of FOLFOX/CAPOX before and after surgery as experimental arm and reported a trend towards better disease-free survival, that was comparable to the effect size in FOXTROT together with a significant effect on overall survival (secondary endpoint) [10]. Given that the FOXTROT trial has reported a trend towards improved efficacy, the PROTECTOR / FIRE‑10 trial will implement a 2 vs. 1 randomization in favor of the preoperative/neoadjuvant therapy algorithm.

In comparison to the FOXTROT and OPTICAL trial, PROTECTOR / FIRE‑10 will exclude dMMR/MSI tumors, as these derived no benefit in the previous trials [9, 10]. Furthermore, we do escalate the duration of perioperative treatment from 6 to 12 weeks in accordance with current concepts in rectal cancer to ensure more efficacy [11, 16]. As an escalated intervention to improve tumor shrinkage – knowing that many patients will present with large primary tumors and the risk for a relevant R1-resection rate will be considerably high [9] – a highly active triplet-regimen (mFOLFOXIRI) [8, 17, 18] is added as treatment option.

Patients fulfilling the eligibility-criteria of PROTECTOR / FIRE‑10 represent a population with relevant risk to develop distant metastases and are consecutively candidates for systemic therapy. Several aspects support the use of systemic therapy prior to a pathological TNM-staging (by resection) of the tumor:

- Earlier treatment (6–8 weeks) of distant micrometastases.
- Increased exposition to chemotherapy as appr. 20% of initially operated patients are not eligible for post-operative chemotherapy (complications, patient preferences) [9].
- Reduction in tumor size, easier resection and likely less complications [9].

On the other hand, it can be argued that pre-operative staging with CT-scans will result in a certain fraction of patients with “over-staging” that consecutively receives chemotherapy in the experimental arm despite a “real” TNM stage that would not qualify for adjuvant systemic therapy if upfront assessed after surgery. Therefore, PROTECTOR / FIRE‑10 will implement control systems to overlook and reduce the proportion of “over-staged” patients:

- Radiology workshop to provide quality control of CT assessments. These will be organized virtually and refer to standard cases and demonstrate criteria of selection of patient for the trial.
- Reassessment of TNM-stage in the control arm after 50, 100 and 150 primarily resected patients and consecutively the option to react to the frequency of over-staged patients - if necessary.

In conclusion, the PROTECTOR / FIRE‑10 trial is designed to provide evidence whether patients – and if so, which patients – will benefit from a preoperative systemic therapy before surgical resection of locally advanced colon cancer including the upper third of the rectum.

### Trial status

Activation of centers started in March 2025 and the first patient was recruited in on March 27th .

Study protocol: Protocol Version 1.1 (Nov. 11, 2024).

## Supplementary Information

Supplementary Material 1.

## Data Availability

Not applicable.

## Abbreviations

CAPOX: Capecitabine plus oxaliplatin
DNA: Deoxyribonucleic acid
ECOG: Eastern cooperative oncology group
FOLFOX: Leucovorin calcium (folinic acid), fluorouracil, and oxaliplatin
FOLFOXIRI: Folinic acid, 5-fluorouracil, oxaliplatin and irinotecan
mFOLFOX6: 5-Fluorouracil, leucovorin, and oxaliplatin
mFOLFOXIRI: Folinic acid, 5-fluorouracil, oxaliplatin, and irinotecan
OS: Overall survival
PFS: Progression free survival
RECIST: Response evaluation criteria in solid tumors
TNT: Total neoadjuvant therapy
XELOX: Capecitabine (Xeloda) and oxaliplatin
